# Moving forward in Rheumatoid Arthritis-Associated Interstitial Lung Disease Screening

**DOI:** 10.3390/jcm13185385

**Published:** 2024-09-11

**Authors:** Javier Narváez

**Affiliations:** Department of Rheumatology, Hospital Universitario de Bellvitge & Bellvitge Biomedical Research Institute (IDIBELL), Feixa Llarga, s/n. Hospitalet de Llobregat, 08907 Barcelona, Spain; fjnarvaez@bellvitgehospital.cat

**Keywords:** rheumatoid arthritis, interstitial lung disease, screening, risk factors, lung ultrasonography

## Abstract

Patients with rheumatoid arthritis (RA) are at increased risk of developing interstitial lung disease compared to the general population, a complication that is associated with significant morbidity and high mortality. Given its frequency and severity, ILD should always be considered during both the initial assessment and follow-up of RA patients. However, there is currently no consensus on which RA patients should be screened for ILD. In recent years, several scientific societies have developed specific screening proposals. According to the recommendations of the Spanish, American, and Austrian rheumatology societies, it is not necessary to screen all individuals with RA, and it should be tailored to each patient based on clinical risk factors. In contrast, the Portuguese Societies of Rheumatology and Pulmonology advocate for systematic screening of all RA patients. Risk factors for the development of ILD in RA patients are well identified, and several screening tools for RA-ILD based on these risk factors have been developed. However, all of these tools still require further validation. To address this issue, the ANCHOR-RA study, a multinational cross-sectional initiative, has been launched to develop a multivariable model for predicting RA-ILD, which could provide valuable guidance for screening practices in clinical settings. In addition to certain biochemical and genetic predictive markers, lung ultrasound appears to be a useful screening tool. When combined with clinical evaluation and risk factor assessment, it can help identify which patients require a thoracic HRCT evaluation, which remains the gold standard for confirming an ILD diagnosis.

## 1. Introduction

Interstitial lung disease (ILD) is one of the most prevalent and severe extra-articular manifestations of rheumatoid arthritis (RA), significantly contributing to both morbidity and mortality [1,2,3,4,5,6,7]. In patients with RA, the most frequent subtypes of ILD are usual interstitial pneumonia (UIP) and non-specific interstitial pneumonia (NSIP). UIP associated with RA-ILD phenotypically resembles UIP associated with idiopathic pulmonary fibrosis (IPF), both of which carry a high risk of progressive pulmonary fibrosis (PPF) and increased short-term mortality. Additionally, some RA patients with NSIP-type ILD may also experience progressive fibrosis [7].

The clinical course of RA-ILD is highly variable (8–10). In some patients, the disease progresses to PPF, characterized by a rapid decline in lung function, rapid progression to chronic respiratory failure, and increased risk of premature mortality. Although RA-ILD can significantly impact patient prognosis, there is ongoing debate about when, whom, and how to screen for this complication. This review examines the current landscape of RA-ILD screening practices, highlights recent advancements, and explores future directions in this area.

## 2. Why Is Screening Necessary? Understanding the Extent of the Problem

ILD can develop at any point during the course of RA. In more than half of the cases, ILD occurs after the diagnosis of RA, typically within the first 5 to 10 years [1,2,3,4,5,6,7,8,9,10]. In these cases, late diagnosis is not uncommon, as lung involvement is often asymptomatic or presents with only mild symptoms in its early stages. Indeed, when all individuals with RA were screened for ILD using thoracic high-resolution computed tomography (HRCT), a significant percentage of subclinical disease was detected (ranging from 11.9% to 55.7%), confirming that this complication is frequently underdiagnosed [11,12]. Less frequently, ILD may manifest at the onset of RA or even precede the joint manifestations by several months or years. Data from our early RA cohort indicate that these situations occur in 32.5% and 17.5% of cases, respectively [13]. In the latter circumstance, RA-ILD is often misdiagnosed as an idiopathic form, despite the differences in prognosis and treatment [1,2,3,6,14].

Although there have been significant improvements in prognosis over the past 25 years [15], ILD remains the second leading cause of death in RA patients, following cardiovascular complications. Approximately 55% of RA-ILD patients experience disease progression [16,17], with an estimated 40% meeting the criteria for PPF within 5 years of onset [18,19]. Patients with RA-ILD have an adjusted mortality risk that is 3- to 10-fold higher than that of RA patients without this complication, regardless of follow-up duration or the presence of comorbidities [4,6,20]. The average survival time following an RA-ILD diagnosis ranges from 2.6 to 8.1 years [5].

## 3. Screening for RA-ILD: Recommendations by Scientific Societies

Rheumatologists play a crucial role in screening patients with RA for ILD. Early detection and assessment of RA-ILD are essential for initiating treatment promptly, as patients may already have significantly impaired lung function at the time of RA-ILD diagnosis. A pivotal study with 167 patients revealed that, at the time of diagnosis, 14% of RA-ILD patients had a forced vital capacity (FVC) below 50% of the predicted value, and 29% had a hemoglobin-corrected diffusing capacity for carbon monoxide (DLCO) below 40% of the predicted value [16]. Another recent study showed that delayed diagnosis of RA-ILD was linked to higher mortality rates [21]. However, there is still no consensus on which RA patients should be screened for ILD.

In the absence of evidence supporting the effectiveness of universal screening, a reasonable strategy is to implement selective screening based on clinical risk factors.

This approach was exemplified by the AR-EPIDSER Project, a collaborative effort between the Spanish Society of Rheumatology (SER) and the Spanish Society of Pulmonology and Thoracic Surgery (SEPAR). The project developed a multidisciplinary proposal for screening criteria aimed at the early identification of patients with RA-ILD [22].

Based on published evidence and using consensus-based techniques (Delphi method), screening for ILD was proposed in three scenarios: (1) patients with a history of respiratory symptoms (cough and/or dyspnea) lasting more than 3 months; (2) patients with dry “velcro-like” crackles on respiratory auscultation, even if asymptomatic; and (3) in patients without respiratory symptoms and with normal respiratory auscultation, screening will be based on the score obtained according to their number of risk factors for developing this complication [22]. Details of the proposal and the frequency of screening are shown in Table 1 and Table 2.

This initiative is based on the consensus of a national panel of experts, and its effectiveness in identifying these patients in clinical practice will need to be confirmed through future validation studies. To address this, a multicenter study sponsored by the SER is currently underway to evaluate its clinical utility. While awaiting the results, which may take some time to become available, we conducted an external validation study of the AR-EPIDSER criteria in our cohort of early RA patients diagnosed between 2003 and 2023 [23]. In all cases, systematic screening for ILD was performed at diagnosis using a targeted medical history, respiratory auscultation, chest X-ray (CXR), and pulmonary function tests (PFTs), including %pFVC and %pDLCO. In cases of respiratory symptoms, velcro-like dry crackles, or abnormalities on CXR or PFTs, a thoracic HRCT was performed. Of the 146 patients included, 28 (19.1%) were finally diagnosed with ILD by HRCT. Ninety patients (61.6%) met the AR-EPIDSER screening criteria for ILD at the time of RA diagnosis. Among these patients, 28.8% had either clinical or subclinical ILD. Of the 56 patients who did not meet the screening criteria at the onset of RA, only 1.3% developed ILD during follow-up. The sensitivity of the criteria in our cohort was 92.8%, and the specificity was 45.7% (data pending publication). In 12 patients, ILD preceded the onset of joint symptoms. Excluding these cases, where screening would not have been necessary, the sensitivity was 87.5%, while specificity remained at 45.7%. If the threshold is lowered to a score of ≥4 (instead of the cut-off of ≥5 established in the original document), sensitivity increases to 90.91%, but specificity decreases to 23.8%.

Based on these results, the AR-EPIDSER criteria for ILD screening demonstrate a sensitivity greater than 90% in patients with early RA, supporting their use in daily clinical practice. Given that the primary goal of screening is the early detection of this complication, it is essential to employ a highly sensitive test in the initial phase to identify as many cases as possible. This approach is consistent with the recommendations outlined in the policy framework for population screening by the Public Health Committee of the Spanish Ministry of Health [24].

After the publication of the AR-EPIDSER criteria, various scientific societies drafted specific recommendations for ILD screening in patients with RA. Consistent with the SER/SEPAR proposal, neither the American College of Rheumatology (ACR) nor the Austrian Society of Rheumatology recommends universal screening for ILD in RA patients. The recently published 2023 ACR/American College of Chest Physicians (CHEST) Guideline for the Screening and Monitoring of ILD in People with Systemic Autoimmune Rheumatic Diseases emphasizes that screening should be tailored to individual RA patients at high risk for ILD [25]. Risk factors that may warrant screening include high titers of rheumatoid factor (RF) or anti-cyclic citrullinated peptide antibodies (ACPA), cigarette smoking, older age at RA onset, and high disease activity. Screening should be conducted using PFTs and thoracic HRCT. If the initial screening is negative, it may be repeated annually. If ILD is confirmed, it is recommended to repeat PFTs every 3 to 12 months during the first year and adjust the frequency thereafter based on the patient’s clinical progression.

In the recommendations of the Austrian Society of Rheumatology, developed through a Delphi consensus, no agreement was reached on the specific combination of risk factors that would justify screening in asymptomatic patients [26]. The panel concluded that there is currently insufficient evidence to support any specific scoring or weighting of these risk factors. Consequently, they left the decision to the physician’s discretion, emphasizing that the assessment of risk factors and the decision to initiate continuous RA-ILD screening should be made on a case-by-case basis.

In contrast, the recommendations of the Portuguese Societies of Rheumatology and Pulmonology [27] advocate for systematic screening of all RA patients using PFTs and CXR. If abnormalities are detected, a thoracic HRCT should be conducted. The frequency of reevaluation depends on the presence or absence of risk factors. For high-risk patients, it is recommended to perform PFTs annually and HRCT every two years. In low-risk patients, PFTs should only be repeated if symptoms develop.

## 4. Tools for Estimating the Risk of ILD in RA Patients

The main recognized risk factors for the development of ILD in RA include male sex, older age, late disease onset, duration of RA, tobacco exposure, moderate or high sustained RA activity, seropositivity for RF and/or ACPA, and polymorphisms in the MUC5B gene [4,7,15,22,28]. In addition to the Delphi panel AR-EPIDSER proposal, several RA-ILD screening tools based on these risk factors have been developed. However, all of these tools require further validation.

Paulin et al. [29] proposed a risk score model based on five variables: male sex (1 point), smoking status (2 points), the presence of extra-articular manifestations (1 point), Clinical Disease Activity Index (CDAI > 28: 1 point), and erythrocyte sedimentation rate (ESR > 80 mm/h: 4 points). A total score of 2 points yields a sensitivity of 90.3% and a specificity of 63.64% for identifying RA-ILD. When the score increases to 4 points, sensitivity decreases to 51.9% while specificity rises to 90.9%.

A simpler risk estimation model, the Four Factor Risk Score, was proposed by Koduri et al. [30]. This model is based on 4 risk factors: smoking (current or past), advanced age, RF, and ACPA. By incorporating these factors, a risk score system was developed using multivariate logistic regression models, which classifies patients as low- or high-risk on a scale of 0 to 9 points. A threshold of 5 points provides a sensitivity of 86%, a specificity of 85%, and an area under the curve (AUC) of 0.76 (Table 3).

Another model, which incorporates factors such as sex, tobacco exposure, RF, C-reactive protein, and plasma matrix metalloproteinase (MMP)-3, showed a C-index of 0.826 for accuracy in detecting RA-ILD compared to evaluations conducted by a multidisciplinary team [31].

The VECTOR algorithm, designed to detect velcro-like crackles in lung sounds recorded by an electronic stethoscope, demonstrated 93% sensitivity and 77% specificity for identifying ILD on HRCT in RA patients [32]. However, the availability of such equipment is limited.

A predictive score based on sex, age at RA onset, the RA Disease Activity Score in 28 joints using ESR (DAS28-ESR), and the MUC5B rs35705950 risk allele showed 75% sensitivity and 85% specificity for identifying RA-ILD when compared to HRCT [33] but this tool may be challenging to implement in clinical practice.

Finally, the VARA-ILD Combined Risk Score model was developed using data from a North American cohort of veterans with RA. This model integrates five single nucleotide polymorphisms (MUC5B, DSP, LRRC34, OBFC1, and FAM13A) along with male sex, age, smoking status, disease activity assessed by DAS-CRP, and RF positivity, achieving an AUC of 0.67 [34].

The ANCHOR-RA study is a multinational, cross-sectional study designed to create a multivariable model for predicting RA-ILD, which could be applied to guide screening for RA-ILD in clinical practice [35]. The study will enroll 1200 participants from USA, UK, Germany, France, Italy, and Spain, all of whom will present with at least two risk factors for ILD, such as male gender, smoking history, older age at RA diagnosis, or high disease activity. This study will assess the prevalence of RA-ILD and investigate the effectiveness of lung ultrasound as a screening tool, comparing its accuracy to HRCT for detecting ILD.

## 5. Biochemical and Genetic Markers

In the search for biochemical markers to predict the development of ILD in RA, neither Krebs von den Lungen 6 (KL-6) nor anticarbamylated protein antibodies have demonstrated predictive value superior to that of ACPA or RF [36,37,38].

Preliminary data suggest that angiogenic T cells could serve as a useful biomarker for this purpose [39]. Additionally, findings from a recent multicentre, prospective RA cohort study have revealed that elevated concentrations of MMPs, particularly MMP-7 and MMP-9, are associated with both the presence of RA-ILD and an increased risk of developing incident ILD [40]. This association was especially strong for MMP-7, with participants in the highest quartile of MMP-7 concentrations having nearly fourfold increased odds of prevalent ILD and a twofold increased risk of incident ILD, typically developing within an average of four years following cohort enrolment. Notably, higher plasma MMP-7 concentrations were predominantly observed in cases of prevalent RA-ILD with a UIP pattern, while MMP-9 showed a modest correlation with impaired %pFVC. In recent years, accumulating clinical evidence has supported the role of MMPs in the pathogenesis of RA-ILD, independent of articular disease activity [40,41].

Regarding genetic biomarkers, the gain-of-function MUC5B rs35705950 promoter variant has been identified as a significant risk factor for both IPF and RA-ILD with a UIP phenotype [42]. Additionally, mutations in telomere-related genes such as DSP, LRRC34, OBFC1, and FAM13A, which are associated with accelerated telomere shortening, have also been implicated [34]. As previously mentioned, these genetic factors are beginning to be incorporated into predictive risk models [33,34].

## 6. Usefulness of Lung Ultrasound

The role of lung ultrasound in screening for ILD in various systemic autoimmune rheumatic diseases, including RA, has been increasingly investigated in recent years. This technique is based on interpreting findings related to changes in the lung’s physical properties, which are detected as artifacts rather than anatomical structures. Studies assessing the usefulness of lung ultrasound for ILD screening have primarily focused on evaluating B-lines and the pleural line. More recently, research has begun to explore diaphragm function [43,44].

The B lines are reverberation artifacts associated with septal thickening. They appear as well-defined hyperechoic vertical lines that begin at the pleural line and extend to the bottom of the screen without fading. B lines, also referred to as “comet-tails,” are artefacts that occur when the air content in the pulmonary parenchyma partially decreases and/or the interstitial space expands in volume. They move synchronically with lung sliding. B lines are not exclusive to ILD, as they can also be observed in other conditions such as pulmonary oedema, and they do not allow for differentiation between the inflammatory or fibrotic phases of ILD [43,44]. The presence of multiple B-lines is a key ultrasound indicator of lung interstitial syndrome. In 2012, efforts to standardize this finding resulted in a consensus definition, establishing the diagnostic criterion as the detection of three or more B-lines in at least two areas on each side of the chest [45]. Pleural line abnormalities include irregularities, thickening, microscopic consolidation, fragmentation, and subpleural nodules. The cut-off for considering a pleural line as thickened is generally set at 2.4 mm, although some authors suggest 2.8 mm [43,44]. For diaphragm evaluation, it is necessary to assess diaphragmatic dysfunction, inspiratory thickness, expiratory thickness, and the thickening fraction.

The strongest evidence supporting the use of lung ultrasound has been published in systemic sclerosis (SSc), where the assessment of appearance, criterion, and construct validity is more advanced, both in the early and advanced stages of the disease. In this context, a strong correlation has been observed between B lines and thoracic HRCT or PFTs (including %pFVC and %pDLCO) [43,44]. In terms of pleural line evaluation, some researchers suggest it has a higher negative predictive value for ILD than B lines and better differentiation from healthy controls [44].

To date, seven published studies [46,47,48,49,50,51,52] have evaluated the usefulness of pulmonary ultrasound in screening for ILD in RA (see Table 4). In cases where ILD is suspected, mainly because of respiratory symptoms and/or dry crackles on auscultation, the sensitivity of pulmonary ultrasound compared with thoracic HRCT ranges from 62.2% to 98.3%. Specificity ranges from 14.7% to 97.6%, positive predictive value from 42.2% to 88.4%, and negative predictive value from 69.5% to 87.5%. When the technique is used to detect asymptomatic or subclinical DILD, sensitivity ranges from 90.6% to 97.1%, specificity from 73% to 97.3%, positive predictive value from 59.2% to 94.3%, and negative predictive value from 94.7% to 98.6%.

Thus, lung ultrasound has proven to be a valuable tool for systematic screening of ILD in patients with RA, demonstrating very high sensitivity and negative predictive value. It serves as a useful complement to clinical information in identifying patients who are candidates for thoracic HRCT, which remains the gold standard for confirming the diagnosis of ILD. Moreover, Otaola et al. [46] found the diagnostic sensitivity of pulmonary ultrasound to be higher than that of PFT and of dry crackles on auscultation.

Major limitations of the technique include the considerable heterogeneity in the published evidence, particularly regarding B line echographic counts and indexes, the cut-off points set to define the disease, and the types of equipment used (from high-range ultrasound devices to pocket-size devices), probes (cardiac, linear, or convex), and the examiner’s experience. Since the first index based on 72 intercostal spaces, counts have gradually decreased to 8 intercostal spaces in the search for easier performance while maintaining precision. However, there is still no consensus on the learning curve, procedural steps, or validated index needed to ensure its effective and reliable use in clinical practice.

## Figures and Tables

**Table 1:** The AR-EPIDSER Project: proposed screening criteria for interstitial lung disease in patients with rheumatoid arthritis [22].

ILD Screening Will Be Conducted in These Three Clinical Scenarios	
(1) Patients with respiratory symptoms (such as cough and/or dyspnea) lasting for more than 3 months.	
(2) Patients with dry, “velcro-like” crackles on respiratory auscultation, even if they are asymptomatic.	
(3) For patients without respiratory symptoms and with normal respiratory auscultation, screening will be based on the score calculated from the number of risk factors present for developing this complication. Any patient with a score of ≥ 5 points will be considered eligible for screening.	
***List of variables and the suggested score for each variable used in the overall calculation***	***Score***
Older age (≥60 years)	2
Male sex	1
Tobacco exposure (active or ex-smoker)	
≤20 pack-years	2
>20 pack-years	3
RA duration of more than 5 years	1
Persistent moderate to high disease activity: an average DAS28-ESR > 3.2 from the time of diagnosis in early RA (defined as symptom duration ≤ 12 months) or a DAS28-ESR > 3.2 for at least 6 months in established RA	1
Serology (only the criterion with the highest weighting is counted towards the total score):	
RF positive > 3 times the ULN	1
ACPA-positive ≤ 3 times above the ULN	2
ACPA-positive > 3 times the ULN	3
Family history of ILD	1
**Screening approach** For patients with cough and/or dyspnea >3 months, start with CXR and PFTs (spirometry, %pDLCO). Based on results, consider thoracic HRCT *	
For patients with dry ‘velcro-like’ crackles on auscultation, perform HRCT directly. In asymptomatic patients with normal auscultation, if the risk score is 5–6, start with CXR and PFTs (spirometry and %pDLCO); consider thoracic HRCT based on results *. If score ≥7, perform HRCT directly	

*Abbreviations:* ACPA: anti-cyclic citrullinated peptide antibodies; CXR: chest X ray; DAS: Disease Activity Score; HRCT: high-resolution computed tomography; ILD: diffuse interstitial lung disease; %pDLCO: predicted diffusing capacity for carbon monoxide corrected for hemoglobin; PFTs: pulmonary function tests; RA: rheumatoid arthritis; RF: rheumatoid factor; ULN: upper normal limit. * If PFTs are unavailable or there is a long waiting list, consider thoracic HRCT directly to expedite diagnosis. Direct HRCT doesn’t exclude PFTs for ILD severity assessment.

**Table 2:** Frequency of screening for interstitial lung disease in patients with rheumatoid arthritis according to the recommendations of the AR-EPIDSER Project [22].

Frequency of Screening
During the follow-up of RA patients, auscultation should be performed at least annually, along with specific questioning about respiratory symptoms and an assessment of risk factors for ILD, based on the scoring system outlined above. If ‘Velcro-like’ crackles or respiratory symptoms (cough and/or dyspnea >3 months) are detected during follow-up, repeat screening tests as recommended, regardless of prior negative results For asymptomatic patients with normal respiratory auscultation and a total score of ≥5, repeat screening tests (including spirometry and %pDLCO) after one year, even if the initial results are negative.

*Abbreviations:* ILD: diffuse interstitial lung disease; %pDLCO: predicted diffusing capacity for carbon monoxide corrected for hemoglobin.

**Table 3:** Four Factor Risk Score model [30].

Score			
	0	1	2
Age at onset of RA	<40	40–70	>70
Tobacco exposure	Never	Ex-smoker or current smoker	
RF titer	Negative	Weak positive	Positive
ACPA titer	Negative	Weak positive	Positive

*Abbreviations:* ACPA, anti–citrullinated peptide antibody; RA: rheumatoid arthritis; RF: rheumatoid factor.

**Table 4:** Studies on the usefulness of lung ultrasound in detecting interstitial lung disease in patients with rheumatoid arthritis.

	Number of Patients	Population	Number of Intercostal Spaces Evaluated	Diagnostic Criteria for ILD	Results (Compared with Chest HRCT)
Cogliati C et al. [46]	39 RA	Suspected ILD	72 and 8	72 IS >17 B-lines 8 IS >10 B-lines	*8 IS >10 B-lines* Sensitivity 69% Specificity 88% *72 IS >17 B-lines* Sensitivity 92% Specificity 56%
Moazedi-Fuerst FC et al. [47]	64 RA and 40 healthy controls	No respiratory symptoms, normal PFTs findings	18	B-lines in ≥2 chest areas Pleural thickening >2.8 mm and at least 1 subpleural nodule	Sensitivity 97.1% Specificity 97.3% PPV: 94.3% NPV: 98.6%
Otaola M et al. [48]	106	No respiratory symptoms (ILD detected by thoracic HRCT in 32)	14	≥5 B-lines	Sensitivity: 90.6% Specificity: 73% PPV: 59.2% NPV: 94.7% AUC: 0.82 *PFTs* Sensitivity %pFVC: 28.1% Specificity %pDLCO: 63.3% *Crackles on auscultation* Sensitivity: 68.8%
Santos Moreno P et al. [49]	192	Respiratory symptoms and/or crackles on auscultation	72	>11 B-lines	Sensitivity: 98.3% Specificity: 14.7% PPV: 64.2% NPV: 84.6% AUC: 0.63
Mena Vázquez N et al. [50]	71	35 with ILD and 36 without ILD	72 y 8	72 IS > 5 B-lines 8 IS > 5 B-lines	A 8-space reduced score showed a similar total predictive capacity than 72-space score. *8 IS >5 B lines* Sensitivity: 62.2% Specificity: 91.3% PPV: 88.4 NPV: 69.5%
Di Carlo M et al. [51]	72	Suspected ILD	14	>9 B-lines	Sensitivity: 70% Specificity: 97.6% AUC: 0.83 Positive likelihood ratio of 29.4
Sofíudóttir BK et al. [52]	77	Respiratory symptoms	14	≥10 B-lines or pleural line abnormalities (thickening and fragmentation)	Sensitivity: 82.6% Specificity: 51.9% PPV: 42.2% NPV: 87.5%

*Abbreviations:* AUC: area under the curve; HRCT: high-resolution computed tomography; ILD: interstitial lung disease; IS: intercostal space; NPV: negative predictive value; %pDLCO: predicted diffusing capacity for carbon monoxide corrected for hemoglobin; %pFVC: predicted forced vital capacity; PFTs: pulmonary function tests; PPV: positive predictive value.
